# Co-Creation of a Multi-Component Health Literacy Intervention Targeting Both Patients with Mild to Severe Chronic Kidney Disease and Health Care Professionals

**DOI:** 10.3390/ijerph182413354

**Published:** 2021-12-18

**Authors:** Marco D. Boonstra, Sijmen A. Reijneveld, Gerjan Navis, Ralf Westerhuis, Andrea F. de Winter

**Affiliations:** 1 Department of Health Sciences, University Medical Center Groningen, 9713 GZ Groningen, The Netherlands; s.a.reijneveld@umcg.nl (S.A.R.); a.f.de.winter@umcg.nl (A.F.d.W.); 2 Department of Nephrology, University Medical Center Groningen, 9713 GZ Groningen, The Netherlands; g.j.navis@umcg.nl (G.N.); r.westerhuis@dcg.nl (R.W.)

**Keywords:** chronic kidney disease, health literacy, self-management, communication, patient education, intervention, professional training

## Abstract

Limited health literacy (LHL) is common in chronic kidney disease (CKD) patients and frequently associated with worse self-management. Multi-component interventions targeted at patients and healthcare professionals (HCPs) are recommended, but evidence is limited. Therefore, this study aims to determine the objectives and strategies of such an intervention, and to develop, produce and evaluate it. For this purpose, we included CKD patients with LHL (*n* = 19), HCPs (*n* = 15), educators (*n* = 3) and students (*n* = 4) from general practices, nephrology clinics and universities in an Intervention Mapping (IM) process. The determined intervention objectives especially address the patients’ competences in maintaining self-management in the long term, and communication competences of patients and HCPs. Patients preferred visual strategies and strategies supporting discussion of needs and barriers during consultations to written and digital strategies. Moreover, they preferred an individual approach to group meetings. We produced a four-component intervention, consisting of a visually attractive website and topic-based brochures, consultation cards for patients, and training on LHL for HCPs. Evaluation revealed that the intervention was useful, comprehensible and fitting for patients’ needs. Healthcare organizations need to use visual strategies more in patient education, be careful with digitalization and group meetings, and train HCPs to improve care for patients with LHL. Large-scale research on the effectiveness of similar HL interventions is needed.

## 1. Introduction

At least 25% of chronic kidney disease (CKD) patients have limited health literacy (LHL) [1], which is associated with faster kidney function decline and higher mortality [2,3,4]. Health literacy (HL) is ‘the degree to which people are able to access, understand, appraise and communicate information to engage with the demands of different health contexts to promote and maintain good health across the life course’ [5]. To improve the health outcomes of CKD patients with LHL, it is necessary to tailor healthcare to their needs [6].

Patients with LHL often have insufficient self-management capacities [7,8,9]. Self-management is defined as the ability to manage the symptoms, treatment, physical and psychosocial consequences, and lifestyle changes inherent in living with a chronic condition. Efficacious self-management encompasses the ability to monitor one’s condition and to affect the cognitive, behavioral and emotional responses necessary to maintain a satisfactory quality of life [10]. Interventions directed at patients have been proven effective at optimizing self-management [10].

To support self-management, healthcare professionals (HCPs) not only need to provide patients with information, but also help them to build the confidence and skills to fulfill self-management activities [11]. However, several communication barriers between HCPs and patients with LHL exist that may hinder effective self-management. For example, patients are less able to discuss their needs and have problems retrieving and understanding information provided during consultations [12,13]. HCPs often fail to recognize patients’ LHL, tend to overestimate patients, and lack the competence to mitigate HL-related problems effectively [14,15].

Recently, we uncovered multiple additional self-management and communication barriers related to LHL that are CKD specific. These are the starting point of the intervention development in this study. Details are in Supplemental Table S1. In brief, the main barriers are that HCPs have problems responding to HL problems and provide limited information when patients experience mild to moderate CKD. Patients with severe or end-stage CKD consider information to be overwhelming, and self-management of lifestyle and medication to be complex. Finally, patients report problems in maintaining better self-management in the long term [16]. The long-term effect of existing HL interventions, targeting self-management behaviors of CKD patients with LHL, often with digital strategies, is unknown [17]. These interventions do not aim to increase competences of HCPs and target groups often were not consulted during development [18,19,20,21,22,23,24,25].

However, multi-component and co-created interventions, aiming to establish more productive interactions between patients with LHL and HCPs [26], are more likely to be accepted or effective [27,28,29,30]. Multi-component interventions have several elements which all contribute to establish change [31]. In such interventions, for example, separate components aim to improve the patient’s knowledge, health behaviors, or the communication competences of HCPs. In our study, co-creation is approached from two perspectives. Firstly, we aim to co-create aligned intervention components targeting both patients and HCPs to facilitate patient-centered communication, which is central in definitions regarding co-creation of care [31]. Secondly, we apply several co-creation methods during the defining, production and evaluation of the intervention (i.e., interviews and usability tests). Co-creation is defined as a participatory approach in cooperation between researchers and target groups, to ensure interventions meet their needs, preferences and abilities with an understanding of the specific context and setting [27]. To our knowledge, no co-created and multi-component HL interventions targeting both CKD patients with LHL and HCPs exist.

It is unclear how the objectives, strategies and content of such an intervention can be aligned to the needs of LHL patients with CKD and HCPs. To overcome this problem, we combined the principles of the Intervention Mapping (IM) protocol and co-creation to develop an intervention step by step, targeting the self-management and communication competences of CKD patients with LHL and the competence of HCPs in supporting these patients. Firstly, we aimed to determine the objectives and strategies for the intervention. Secondly, we aimed to design and produce the intervention and evaluate if it was usable, comprehensible, and met the needs of the target groups.

## 2. Materials and Methods

In this study, we followed the IM protocol, which describes an iterative process to develop interventions by (1) analyzing the needs and problems of the target group, (2) formulating change objectives, determinants and expected outcomes, (3) generating and developing theory- and evidence-based intervention strategies, (4) translating these into a produced intervention program, and (5) evaluating adoption (i.e., usability, comprehensibility, fit to the needs) and potential implementation barriers of the intervention [32]. Within the steps of the IM protocol, we applied mixed methods to systematically co-create the intervention. The Medical Ethical Committee of the University Medical Center, Groningen (UMCG) approved the study (number: 201900259).

### 2.1. Participant Eligibility and Recruitment

We included Dutch CKD patients with LHL (*n* = 19) and HCPs (*n* = 15) from general practices and nephrology clinics, as well as educators (*n* = 3) and students in nursing and medicine (*n* = 4) from two universities and two nephrology clinics. These participated in at least one step of our study. Our recruitment started with the HCPs, educators and students. They received an e-mail about our research, which asked consent for participation. In each GP and nephrology clinic, one HCP became a contact person. This HCP supported the inclusion of additional HCPs via snowball sampling, and approached patients to include in steps 2 and 3 of the IM protocol. Patients were eligible if they (1) were adult, (2) experienced >3 months of CKD, stages 2–5, and (3) had LHL, measured with the All Aspects of Health Literacy Scale [33]. Major cognitive problems and terminal illness were reasons for exclusion. The HCPs received the eligibility criteria and a checklist, explaining signs of LHL, based on scientific evidence [34,35].

Firstly, the HCPs approached 46 eligible patients by phone or during consultations and provided them with an information letter. Secondly, the first author provided patients with further information; 19 patients were eligible and included. In steps 2 and 3, it became clear that the problems, needs and context of dialysis patients greatly differed from patients in ambulatory settings (CKD-stages 2–4). The intervention produced in step 4 was targeted towards ambulatory setting. Therefore, we excluded the eleven dialysis patients from that moment. Of the remaining eight eligible patients, four patients dropped out because of SARS-COVID-19 anxiety (*n* = 2), severe illness (*n* = 1), and losing interest (*n* = 1).

### 2.2. Study Procedure

**Figure 1:**
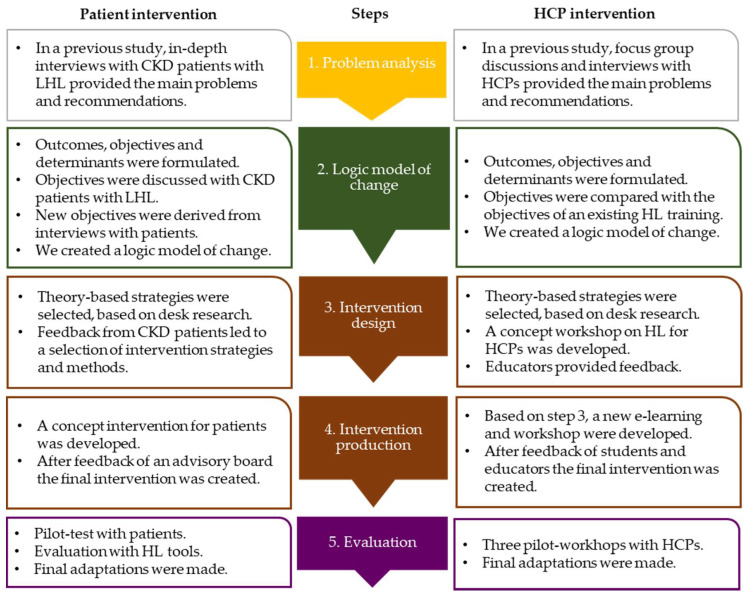
The Intervention Mapping process to develop an intervention for both kidney patients with limited health literacy and health care professionals. CKD = chronic kidney disease, HCP = health care professional, HL = health literacy, LHL = limited health literacy.

The study procedure consisted of a number of steps (Figure 1), described hereafter. Although the steps to develop the patient and HCP intervention were conducted simultaneously to align their components, we will first describe the procedure for developing the patient intervention and then that for developing the HCP intervention.

Step 1: Problem analysis

For both interventions, step 1, the problem analysis, came from the identified barriers for self-management and the proposed solutions in a previous longitudinal, qualitative study, as described in the introduction (see Supplemental Table S1) [16]. This provided the starting point for our intervention development.

#### 2.2.1. Procedure for the Patient Intervention

Step 2: Logic Model of Change

Derived from step 1, the research team formulated preliminary objectives and determinants to intervene in self-management of patients with LHL. Next, an advisory board, consisting of two CKD patients, a medical doctor, a nurse and two researchers provided feedback in a 2-h meeting on these objectives and determinants. With the mixed methods below, we further checked and improved the objectives and determinants to be addressed, and outcomes to be aimed at, to include in a final logic model of change (see Section 2.2.3).

Firstly, we interviewed CKD patients with LHL (*n* = 19) for about 1.5 h each, directly after a consultation with their HCP. We asked open-ended questions on their experiences with self-management and during the consultation to identify new objectives and determinants. Secondly, we asked them to respond to quotations that reflected the preliminary objectives and determinants. After each meeting, we filled in a standardized form to note important experiences, based on rehearsals of the audio recordings of the interview. Examples of the used quotations and the form are in Supplementary Files S2 and S3.

Step 3: Program design

To start developing the patient intervention, we performed desk research. This was intended to retrieve useful theories that could help to organize the objectives and determinants into intervention components, and to identify intervention methods and strategies that fitted our objectives and determinants.

Next, we asked the same patients in step 2 to comment on the identified frequently used intervention methods, such as individual and group counselling, or digital, written and visual communication. During this feedback session, we also showed patients real examples of strategies, and asked for feedback and preferences. These strategies are normally used by several nephrology clinics, the Dutch Kidney Foundation and the Dutch Kidney Patient Association.

Step 4: Program production

In the fourth step, we produced a first draft of the patient intervention. During the production, we linked the final objectives and determinants from step 2 with the strategies from step 3 and developed the content of each component of the intervention. Below we provide a description of the production of this program.

Firstly, a draft version of the intervention was fully designed by the research team in cooperation with a professional graphic designer. Secondly, the advisory board provided feedback on the draft version in a 2-h workshop, and in an additional round of written comments. Thirdly, we improved the draft intervention and prepared it for the evaluation below.

Step 5: Evaluation

The fifth, final step consisted of pilot tests to evaluate the intervention’s adoption and barriers for future implementation. This was done by determining its usability, usefulness, comprehensibility, and fitness for needs.

Firstly, CKD patients with LHL (*n* = 4) used the intervention independently or with the help of a significant other. Secondly, in an interview and evaluation questionnaire, we asked their opinion on the intervention. The recordings of the interviews were analyzed afterwards. Thirdly, independent raters, a researcher from our department and two students in nursing filled in checklists of the Health Literacy Assessment Tool for Identifying Facilitating Factors and Barriers to Information, Care, and Services [36] to check the writing style, organization and design of the patient intervention.

#### 2.2.2. Procedure for the HCP Intervention

Step 2: Logic model of change

Firstly, we formulated preliminary objectives and determinants for the HCP intervention, based on the previous longitudinal, qualitative study [16]. One researcher (M.D.B.) compared the preliminary objectives and determinants with those of a developed and pilot-tested health literacy training program by Kaper et al. [37,38]. Its objectives and determinants are in Supplementary Table S4. Secondly, he discussed the identified similarities and contrasts with a second researcher (A.F.d.W.). This discussion yielded a final set of determinants and objectives for the HCP intervention, which were also added to the logic model of change (see Section 2.2.3).

Step 3: Program design

Based on the final set of determinants and objectives for HCPs, we developed a draft workshop for HCPs. We shared the content of this workshop with two educators to provide feedback on content, methods and chosen strategies. They also checked if the objectives were realistic and applicable to a nephrology context. Both provided feedback with comments on the presentation, which were discussed during a 1-h meeting. This led to a final selection of content, methods and strategies.

Step 4: Program production

An improved draft version of the intervention was produced in three steps. Firstly, based on the comments in step 3, the researchers developed an e-learning program and workshop. Secondly, educators and students provided feedback in 30–60 min meetings and in writing. Thirdly, we analyzed the feedback, and prepared the intervention for the evaluation below.

Step 5: Evaluation

To pilot test the intervention, HCPs (*n* = 15) were invited for a 2-h test session of the HL training. Directly after the training, they filled in a questionnaire with evaluative questions on content and satisfaction, and on the expected effects on HL knowledge, self-efficacy and communication competences.

#### 2.2.3. Synthesis of the Results

The results of steps 2 and 3 for both the patient and HCP intervention were combined in a final logic model of change. Based on this model and the results from step 4 and 5, the final multi-component intervention for both CKD patients with LHL and HCPs was produced.

### 2.3. Measures

Interview Guides

We used interview guides with closed and open-ended questions to facilitate data collection among patients in steps 2 and 5. For step 2, the guide followed the framework of Haes and Bensing [39]. We asked about experiences regarding various consultation aims: (1) fostering the relationship, (2) gathering information, (3) information provision, (4) decision making, (5) enabling disease- and treatment-related behavior, and (6) responding to emotions. For step 5, the guide contained questions about usability, usefulness, comprehensibility and satisfaction with the intervention.

Questionnaires

For patients, during step 2, we administered the AAHLS questionnaire to determine HL level [33]. Patients answered ten 3-point Likert scale items with a score of 1, 2 or 3 per item, giving a maximum possible score of 30. Patients were considered to have LHL when they scored ≤25. Additionally, we asked questions on background characteristics, such as gender, age, education and co-morbidities. The evaluation questionnaire in step 5 encompassed closed 3- or 5-point Likert scale questions on usability, usefulness, comprehensibility and satisfaction. It also had two open questions to inform on the strengths and weaknesses of the intervention.

For HCPs, the evaluation questionnaire in step 5 contained 7-point Likert scale questions to estimate intervention effects on HL knowledge, self-efficacy and competences, and satisfaction with the training, derived from Kaper et al. [37]. At this step, HCPs also filled in questions on their age, gender and professional background.

## 3. Results

### 3.1. Background Characteristics

The 19 patients had a mean age of 69.1; 36.8% were female. Their mean total HL score was 20.7 ± 2.9 and the critical HL score was 6.2 ± 1.6, indicating that many patients experienced problems in searching for and reflecting on information, a problem for self-management. Among the 22 professionals, the mean age was 42.6 and 95.5% were female. Details on their background characteristics are in Table 1.

### 3.2. Results for the Patient Intervention

#### 3.2.1. Step 2: Logic Model of Change

To start, we formulated five preliminary objectives aiming to improve the knowledge and competences of CKD patients with LHL. Additionally, we decided upon a preliminary objective aiming to strengthen the patient’s social network. Within the objectives aiming to improve the knowledge and competences of patients, we thought there was an important role for HCPs, and therefore added multiple determinants to improve the competence of HCPs in supporting patients with LHL. Table 2 shows the preliminary objectives and determinants.

In the interviews, the experiences of patients receiving ambulatory care and dialysis treatment largely differed, indicating that one intervention for both groups would insufficiently meet their needs and problems. To illustrate, patients with mild to severe CKD mostly recognized that they could benefit from the objectives aiming to improve awareness and knowledge, and to equip them to self-manage at home. In contrast, patients with kidney failure on dialysis believed it was more important to receive support to maintain self-management changes in the long term. Dialysis patients were more outspoken that they needed help from their social network to be able to self-manage. A summary of patient experiences, which during step 4 of the IM protocol led to the decision to target the intervention at the ambulatory setting, is in Table 2 as well.

Additionally, the patients’ experiences indicated missing determinants. We formulated nine new determinants aiming to improve understanding of CKD risks, to enable both patients and HCPs to discuss self-management better during consultations, and to help patients to maintain self-management behaviors in the long term. Six determinants were reformulated to better reflect the patient’s experiences. The final objectives and determinants were aligned and combined with the results of step 2 of the HCP intervention into a final logic model of change, which is found in Section 3.3.

#### 3.2.2. Step 3: Program design

Theory-based approach

In the literature, we encountered different models of behavior change [40,41], but the Stages of Change Model (SoCM), suited the experiences of step 2 best and was used to start our intervention development. The SoCM states that individuals move through different stages before reaching behavior change: precontemplation, contemplation, preparation, action, and maintenance [42].

After discussion, we decided upon four components for the intervention. The first component aims to improve CKD awareness and knowledge— the (pre)contemplation stage. The second intends to improve motivation for and preparation of self-management—the preparation stage. The third aims to teach competences to maintain self-management in the long term—the action and maintenance stages. The fourth component targets the HCPs to develop their competences to support patients with LHL. As our intervention does not target social networks, we divided the determinants related to strengthening social networks over the other components, to ensure that the intervention enabled patients and HCPs to involve the network in the treatment and self-management. We enriched the final logic model of change in Section 3.3 with these additions.

Selection of strategies

With desk research, we selected fourteen example strategies suiting the objectives and determinants. Examples are a video animation, leaflet, group meeting, website texts, a card to prepare consultations, recipes, strategies of goal setting, the use of reminders and peer support.

During the interviews, visual strategies, such as an animation explaining CKD and symbols indicating important CKD symptoms to discuss, received the most positive feedback. Patients stated these helped to make them aware, knowledgeable and to make better decisions. Additionally, patients mentioned that improved communication with HCPs should be part of an intervention, because it offers the chance to ask questions and to discuss their personal situation, which is important to facilitate change and to maintain self-management at home. Patients shared their enthusiasm about a card to prepare consultations and to take home the advice of the HCPs.

Written or internet-based strategies, or group meetings were not preferred often. Patients mentioned lacking reading or digital competence or digital devices. Group meetings were not preferred because patients did not want to visit their health care organizations more often, or they felt that the presence of peers would complicate speaking out.

The quotes below illustrate feedback during the interviews:

Patient with moderate CKD, male, 67 years: *‘I need videos or pictures to understand. I do not read often. If I do, the information will not stick. The video I just saw made things clear’*.

Patient with moderate CKD, male, 81 years, about the card to support consultations: *‘This card can help, because I am older. I sometimes don’t know what to say and I have problems remembering everything’*.

Patient with severe CKD, male, 75 years: *‘Group education is not for me. I would feel uncomfortable, and not contribute much. I prefer to do it by myself or with the help of my wife’*.

#### 3.2.3. Step 4: Program Production

The divergent experiences of patients from different settings (Table 2), led to the decision to target the intervention towards the ambulatory setting. Based on step 2 and 3, we developed:

(1) Component one and two for patients: a website and brochure with many visual strategies, such as animations and photo stories. These consisted of two parts. Part one was intended to meet our aim of improving awareness and understanding of CKD and the importance of lifestyle and medication. Part two aimed to explain lifestyle and medication, and to gain competence in communicating with HCPs effectively.
(2) Component three: a card to improve consultations. This card helps patients to prepare and discuss self-management actions, needs and barriers, and HCPs to summarize information and actions for self-management. This card enables the patient to develop practical competences and helps to maintain self-management changes.

For these strategies we collected more feedback. The advisory board of patients, HCPs and researchers stated that the intervention needed further improvement to support practical competences and maintenance of long-term behavior changes. Additionally, they suggested delivering an additional intervention component after the consultation, so that patients could follow up on the advice of the HCP. Therefore, seven topic-based brochures were added to the intervention. These aimed to provide practical guidance, stimulate help-seeking and prevent relapse. Based on the feedback, we completed the intervention with information that helped to improve competences. Examples are the addition of (1) a video/cartoon that helped patients to recognize unhealthy foods, and (2) cartoons showing how patients can make consultations more effective [43]. Furthermore, text was simplified, shortened and re-organized. Discussion of the card led to the adding or combining of icons, for example on emotions and living with kidney disease.

#### 3.2.4. Step 5: Evaluation of the Intervention

Patients, in general, found the intervention easy to use, useful and comprehensible. They reported that it helped to improve their understanding and also to contribute to consultations with HCPs. Details are in Table 3. Additionally, the interviews informed us on worthwhile improvements. Firstly, patients suggested delivering the intervention in smaller steps to prevent the feeling that they needed to do too much at once. Secondly, if GPs did not discuss CKD during consultations, patients did not see why they needed the intervention. Thirdly, sometimes HCPs did not respond adequately to the filled-in card. We ensured that the workshop taught HCPs about the patient intervention better, so that they understood their role.

Patient with moderate CKD, female, 47 years: *‘I learned a lot from this program. I learned about the functioning of the kidneys, and I think it is good to know what information I should share with the doctor’*.

Patient with moderate CKD, male, 77 years: *‘The general practitioner never extensively discussed my kidney problems. So when I used the intervention, I was wondering to what extent it was for me’*.

The checklists of the Health Literacy Assessment Tool [39] gave a combined rating above 70% for all strategies of the patient intervention. This indicated that the writing style, organization and design of the intervention, in general, met the needs of patients with LHL. The contents of the brochure and website were identical, but the website was rated lower. The reason was the website lacked features, such as a search function, because it was structured as an e-learning tool that patients use step by step. The topic-based brochures contained more text, giving a lower score on writing and organization of information. Details are in Supplementary File S5.

### 3.3. Results for the HCP Intervention

#### 3.3.1. Step 2: Logic Model of Change

For the HCP intervention, we formulated one objective, aiming to improve the competence of HCPs in the application of strategies to communicate better with patients with LHL. This is important as, according to the experiences of patients, effective communication is the key to improved self-management. Within this objective, we formulated three determinants:

1. HCPs have awareness and knowledge of health literacy and its consequences.
2. HCPs know and apply strategies to identify patients with LHL.
3. HCPs know and apply tailored strategies to improve awareness, knowledge and self-management, as indicated behind the objectives in Table 2.

Comparison of our preliminary determinants with those of the training of Kaper et al. [37,38] revealed that there was overlap. For example, we embraced the determinants to make HCPs aware and knowledgeable of the prevalence, risks and impact of LHL, to be able to identify patients with LHL, and to improve their patient-centered communication strategies. For example, we considered it important that HCPs are able to recognize patients with LHL, provide simple information, involve patients in shared decision making and enable their self-management. However, as CKD is a chronic disease, we decided to put more emphasis on strategies to enable maintaining long-term self-management by discussing barriers, emotions, concerns and needs of the patients. We dropped determinants related to the assessment and writing of comprehensible information because we aimed for comprehensible information within the patient intervention.

The final objectives, determinants and expected improved outcomes for both the patient and HCP interventions are in the logic model of change in Table 4.

#### 3.3.2. Step 3: Program Design

For HCPs, we developed a draft of a training with different learning strategies, such as a presentation, videos with patient stories and group discussions. The educators stated that the concept training encompassed relevant topics, and were positive about the objectives, the provided information, videos and incorporated patient stories used to illustrate HL problems. The educators further suggested developing an e-learning course with important theory, and to focus the workshop more on CKD setting and practicing of competences. They suggested showing real consultations and discuss those, and to provide the HCPs with a patient story, asking them to identify both HL and self-management problems.

#### 3.3.3. Step 4: Program Production

For HCPs, we developed an e-learning course and workshop to teach competence in recognizing LHL and communicating with LHL patients. Educators and students suggested improvements for the e-learning course and workshop. For example, content providing simple information on (1) CKD, (2) lifestyle and medication, and (3) causal relations was combined. Since HCPs often have had communication education already, they also advised reducing the amount of information on basic communication strategies. They considered emphasis on CKD- and LHL-tailored strategies, such as explaining lab values and overcoming self-management barriers, more important.

#### 3.3.4. Step 5: Evaluation of the Intervention

HCPs expected the training to improve their knowledge, self-efficacy, and use of strategies to recognize and support patients with LHL. All believed the intervention was comprehensible and fitted their needs. Details are in Table 3. In open comments, multiple HCPs considered the two-hour workshop too short and found it too information-heavy. They suggested making it longer with more room for interaction, for example with group discussions or role-playing.

### 3.4. Synthesis of the Results

Final logic model of change

Based on the combined results of steps 2 and 3 for both patients and HCPs, we created the final logic model of change. The preliminary objectives aiming to improve outcomes for patients were organized according to the SoCM into three intervention objectives to improve the patients’ (1) awareness and knowledge, (2) motivation and preparation, and (3) competence in maintaining self-management in the long term. Within these objectives, we formulated determinants for the patients and HCPs, as they both contribute to the expected outcomes. One objective explicitly targets the support by HCPs, by improving their knowledge of HL and effective strategies to recognize and support patients with LHL. Table 4 shows this model with the final objectives, determinants and the expected outcomes to improve.

Final multi-component intervention

**Figure 2:**
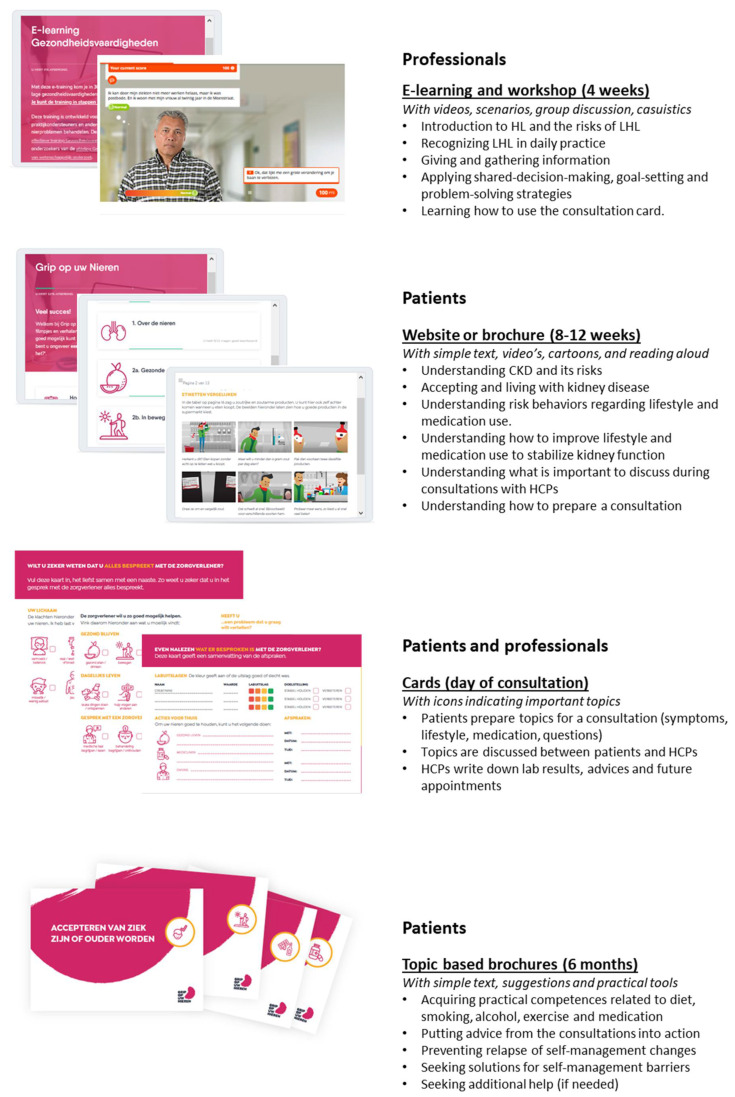
Overview of the final co-created four-component intervention.

Figure 2 gives an overview of the strategies and planning of the final four-component intervention, Grip on your Kidneys. The different components, based on the logic model of change, were planned in detail. The results from steps 4 and 5 provided important input. Patients have 8-12 weeks to use either the website or brochure, and then prepare the consultation. At the same time, HCPs follow an e-learning course and workshop. After the consultation, where the card is used by both the patient and the HCP, patients use the topic-based brochures to learn the practical competence needed for self-management and strategies to maintain self-management in the long term.

## 4. Discussion

Following the principles of the IM protocol, we produced a four-component intervention, Grip on your Kidneys. Three components contain objectives targeting CKD patients with LHL to improve their CKD knowledge and competences to prepare, act upon and maintain self-management. One component has the objective to optimize the competences of HCPs to support these patients. From patients, we learned additional determinants within these objectives, especially to improve their communication competences and maintenance of lifestyle behavior changes. Patients preferred visual strategies, and strategies to improve their contribution to consultations, while HCPs valued training on LHL. Evaluation showed that our intervention is comprehensible and useful for patients with LHL and their HCPs.

The four components of Grip on your Kidneys encompass a comprehensive set of objectives and determinants focusing on both CKD patients with LHL and HCPs. Based on interviews, we added nine determinants targeting their communication competence in discussing self-management needs and barriers and the patient’s competence in maintaining lifestyle changes in the long term. We think these are important to overcome a weakness of HL interventions: that they often focus only on the patients, and specifically their knowledge [30,44]. There is evidence that our final objectives and determinants are relevant. For example, HL theory states it is important to focus on both the patient and HCP to improve self-management and health outcomes [45,46]. Additionally, the importance of teaching the HCPs competences to support patients with LHL is acknowledged [6,14,47]. However, other studies give results challenging our intervention. For GPs, there is evidence that low-intensity interventions have a more positive effect on health behaviors [48], indicating that our intervention objectives might be too extensive. HCPs consider learning competences related to enabling self-management as the most important objective of our training and more relevant, compared to providing simple information [49].

In step 3 of our study, we found that patients with LHL preferred visual strategies and supporting tools to discuss their needs and barriers with HCPs. Written and digital strategies as well as group meetings were not preferred often. Other studies confirm that people with LHL prefer visual strategies, and have problems engaging with digital [50,51], written and oral information, which is also the case in CKD care [45,52,53,54,55]. Our results indicate, as is stated by Brach et al., that healthcare organizations should indeed focus on designing audiovisual information and implementing HL strategies in consultations to improve care for patients with LHL. Our work also shows the relevance of including the patient in the design and evaluation of HL strategies, by using methods of co-creation in the various stages [27,37,56]. In addition to existing knowledge, we show that the type of care, the patient’s competences and disease severity have an influence on the preferred intervention strategies. For example, both the high-intensity dialysis schedule and fear of speaking up for themselves, which is associated with LHL [12,13], hindered the patient’s willingness to visit group meetings. In contrast, patients with mild to severe CKD expected to benefit more from improved consultations, as they felt that they lacked information and some topics were not discussed. Considering our found preferences, it is contradictory that many HL interventions in CKD are web-based [18,23,57]. We show that several patients feel they lack the skills or devices to use these strategies. Our study illustrates the importance of offering intervention flexibility. For example, when patients lack reading skills, they benefit from the website, as text is read aloud. Alternatively, with limited digital skills, a brochure fits better. However, when patients lack both reading and digital skills, or do not speak Dutch, we think our intervention still has shortcomings.

Our pilot testing revealed that the intervention was useful, comprehensible and fit the needs of both patients with LHL and HCPs. It also revealed barriers to implementation, such as the length and accessibility of the intervention, which led to refinements. The added value of the IM protocol mainly lay in the exploration of implementation barriers. Other studies with similar interventions, not developed according to our methods, have limited their attention to the incorporation in daily practice [20,37,47]. Our results indicate that the intervention has the potential to promote self-management among CKD patients with LHL, but a high-quality randomized control trial with a process evaluation is needed to learn about effectiveness and implementation [56].

We successfully combined the IM protocol with co-creation methods to develop Grip on your Kidneys. The protocol sets the framework to determine the objectives and strategies, and to produce and evaluate the intervention. Co-creation methods, within the different steps, heightened the relevance of the intervention and the chance of successful implementation. As the IM protocol does not prescribe the involvement of the target groups in all steps, we advise a combination of those methods to facilitate intervention development.

This study has several strengths. Firstly, our methodology enabled the production of an intervention, which is tailored towards the specific needs of patients with LHL, their HCPs, and the context of CKD care. Secondly, with the AAHLS and recruitment procedure, we managed to include patients with measured LHL, often including those of low education. They are often underrepresented in research and the most important target group for HL research. Thirdly, our evaluation indicated that the intervention was to the satisfaction of the target groups and seemed to have impact on knowledge and competence.

Furthermore, some limitations are worth mentioning. First, only four patients pilot-tested the intervention, which may limit the generalizability of our findings. However, as patients and the advisory board participated in earlier steps and the assessment tools gave good results, we expect our findings to be applicable to CKD settings. Secondly, the intervention for HCPs was only tested with nurses. Despite that, we expect the findings to be generalizable to other CKD professionals, as an evaluation of the training of Kaper et al. yielded similar results among HCPs from different professional backgrounds [38]. Thirdly, it is possible our interviews did not reveal all relevant objectives and determinants. Teasdale et al., for example, mention experiences, such as uncertainty about CKD causation and perceived loss of freedom [58], which were not mentioned by the participants.

Our study has implications for both research and practice. For researchers, we show that the principles of IM and co-creation are useful when developing interventions, for example to uncover unexpected objectives or to select the best strategies. We also illustrate that it is possible to align interventions for patients and HCPs, strengthening their communication, and we invite researchers to develop similar interventions. More HL interventions targeting communication and self-management behaviors need to be researched, preferably using strong intervention designs, such as randomized control trials, in larger groups of participants.

For practice, our study has several implications. Firstly, our study revealed that patients with LHL need improved support to maintain behavior changes and to communicate with HCPs to optimize their self-management. Secondly, organizations need to build the capacities of HCPs to elucidate HL problems, for which our workshop and the work of Kaper et al. offer important objectives and strategies. Thirdly, this study suggests developing more visual strategies, such as animations, to educate patients about the disease and treatment. Fourthly, organizations need to be aware of the risks of digitalization of information and group education. Both strategies are commonly implemented to heighten care efficiency, but might disadvantage patients with LHL or inadequate digital skills. Fifthly, to further refine and evaluate, it is relevant to implement HL interventions, such as Grip on your Kidneys, within different health contexts and settings.

## 5. Conclusions

Supported by the IM protocol and co-creation methods, we developed a comprehensive intervention, Grip on your Kidneys, targeting both CKD patients with LHL and their HCPs. In interviews, we identified multiple important determinants for the intervention, related to communication between patients and HCPs and to needed competences for long-term self-management. A combination of visual strategies, strategies to optimize consultations and training of HCPs was preferred by the target groups to support their self-management. According to our evaluation, the intervention was useful, comprehensible, and met the needs of the target groups.

## Figures and Tables

**Table 1:** Characteristics of patients, health care professionals, educators and students.

Patients (*n* = 19)		Professionals (*n* = 22) ^#^	
**Age**		**Age ^^^**	
mean ± stdev (range)	69.1 ± 12.2 (47–90)	mean ± stdev (range)	42.6 ± 13.0 (21–63)
**Female sex, *n* (%)**	7 (36.8)	**Female sex, *n* (%)**	21 (95.5)
**Educational level, *n* (%)**		**In step 3–4 of IM, profession, *n* (%)**	
Primary education	6 (31.6)	Educator	2 (9.1)
Lower secondary education	4 (21.1)	E-learning educator	1 (4.5)
Lower tertiary education	8 (42.1)	Student Medicine	2 (9.1)
Higher tertiary education	1 (5.3)	Student Nursing	2 (9.1)
**Living situation, *n* (%)**		**In step 5 of IM, profession, *n* (%)**	
Alone	7 (36.8)	**General Practices**	
With partner	12 (63.2)	Specialized nurse	3 (13.6)
**Nationality *n* (%)**		Nurse	1(4.5)
Dutch	17 (89.4)	**Nephrology clinics**	
Other	2 (10.6)	Specialized nurse	1 (4.5)
Type of treatment, *n* (%) *		Nurse	10 (45.5)
Ambulatory (CKD-stage 2–4) ^~^	8 (42.1)	**Working experience *^#^***	
Dialysis (CKD-stage 5)	11 (57.9)	Years, mean±stdev	14.1 ± 10.2
**Co-morbidities, *n* (%)**		(range)	(2–39)
Diabetes	8 (42.4)		
Hypertension	7 (37.1)		
Cardiovascular Diseases	9 (47.7)		
Other	7 (37.1)		
None	2 (10.6)		
**Years of CKD**			
mean ± stdev (range)	14.2 ± 14.3 (1–45)		
**Health literacy (AAHLS)**			
Total HL score, mean ± stdev (range)	20.7 ± 2.9 (13–25)		
Total Funct. HL^+^, mean ± stdev (range)	6.9 ± 1.5 (3–9)		
Total Comm. HL^+^, mean ± stdev (range)	7.6 ± 1.6 (3–9)		
Total Critical HL^+^, mean ± stdev (range)	6.2 ± 1.6 (4–10)		

*n* = the number of participants. stdev = standard deviation. CKD = Chronic Kidney Disease. CKD-stage is based on estimated glomerular filtration rate (eGFR) according to the HCPs who recruited the participants. ^~^ = Patients in ambulatory setting have scheduled consultations about CKD in GPs or nephrology clinics. ^#^ = The group of professionals consisted of educators, students, and health care professionals. AAHLS = All Aspects of Health Literacy Scale. HL = health literacy. Funct. = functional. Comm. = communicative. Maximum possible AAHLS scores: total HL: 30, funct: 9, comm: 9, crit: 12. ^^^ = Calculation based on *n* = 21 because of missing data. IM = Intervention Mapping.

**Table 2:** Preliminary objectives and determinants aiming to optimize the self-management of CKD patients with LHL, and experiences of ambulatory and dialysis treatments according to the in-depth interviews with patients (*n* = 19).

Objective	Determinants	Experiences from Ambulatory Setting	Experiences from Dialysis Setting
Improve CKD awareness	1. HCPs create CKD awareness in LHL patients. 2. Patients are aware of having kidney problems.	**Half of the patients are fully unaware** Patients from GPs (*n* = 4) knew they had proteins in their urine, but were unaware of having CKD. Others had some awareness, but did not consider CKD dangerous.	**Patients are fully aware** All patients (*n* = 11) were fully aware of having CKD and its risks. Two patients stated they became aware when CKD was already severe.
Improve knowledge on CKD and self-management	1. HCPs inform patients in simple language/with visual strategies. 2. HCPs check the patients’ understanding. 3. Patients understand (the symptoms and risks of) CKD. 4. Patients ask questions/clarification from the HCP.	**Patients lack knowledge** More than half of the patients (*n* = 4) lacked knowledge on CKD and CKD self-management. Patients shared problems with reading and understanding information (*n* = 3) and with asking HCPs questions (*n* = 5). The last was related to limited time and space to share personal issues during short consultations.	**Patients struggle with the details** Patients (*n* = 10) knew what CKD is and understood how self-management can stabilize CKD (*n* = 8). However, details on lifestyle and medication were, for many, difficult to understand (*n* = 7). Patients shared problems with reading and understanding information (*n* = 7) and with asking HCPs questions (*n* = 4). Frequent dialysis made asking questions easier.
Improve motivation and preparation of self-management	1. HCPs apply shared decision making to decide on aims of self-management. 2. Patients are intrinsically motivated to self-manage their disease and treatment. 3. Patients share their personal needs regarding self-management with HCPs.	**Not seeing the urgency to self-manage** Half of the patients (*n* = 4) stated lifestyle and medication are important to improve health. Many were not very motivated to make self-management changes for CKD (*n* = 6), because they lacked symptoms or did not know how or why. If patients improved their lifestyle, they often did so because of co-morbidities (*n* = 5). Patients (*n* = 5) felt HCPs were in the lead during consultations.	**Seeing importance, but complicated** All patients (*n* = 11) stated lifestyle and medication are important and knew what they needed to do in their CKD self-management. Negative emotions (*n* = 6), and favoring quality of life over strict adherence (*n* = 6) were reasons not to change lifestyle sometimes. Half of the patients (*n* = 5) felt the HCPs were mainly in the lead in what they needed to do.
Teach competences to self-manage at home	1. HCPs translate general self-management advice into action points. 2. HCPs respond to the patients’ problems. 3. Patients have the practical competence to improve lifestyle and medication.	**CKD self-management is no explicit aim** Few patients (*n* = 3) started to adopt lifestyle changes to stabilize CKD. Most (*n* = 6) gained competence helping them to live healthier in general, as a result of diabetes or hypertension. These patients said advice on lifestyle or medication were not always feasible (*n* = 4).	**Unable to realize all needed changes** All patients claimed to follow up at least some of the lifestyle and medication advice. Half (*n* = 6) said they gained the needed competence. However, it was simply too much, and HCPs do not always succeed in giving realistic advice or help to solve problems (*n* = 7).
Overcome barriers for self- management to maintain behaviors	1. HCPs invite patients to share self-management barriers. 2. HCPs seek for solutions for barriers by applying shared decision making. 3. Patients recognize and solve barriers that negatively influence self-management. 4. Patients know strategies to maintain self-management. 5. Patients share their barriers and concerns with HCPs.	**CKD self-management is no explicit aim** Patients from GPs (*n* = 3) said they did not receive specific self-management advice to stabilize CKD. However, patients (*n* = 6) experienced barriers to self-management on a daily basis, either for diabetes, cardiovasular disease or CKD. Temptations (*n* = 5), lack of rewards (*n* = 2), age or mental problems (*n* = 3) were reasons to give up on self-management. Half of the patients (*n* = 4) felt that barriers were not discussed often.	**Many barriers to maintaining changes** All patients (*n* = 11) shared barriers in the maintenance of self-management. The burden of dialysis (*n* = 2), age or mental problems (*n* = 4), and the fact that their kidneys will never get better (*n* = 5), are all reasons to give up on self-management. Half of the patients (*n* = 6) felt that barriers were not discussed often.
Strengthen the social network	1. HCPs involve the social network in consultation and treatment. 2. HCPs empower the social network to contribute to self-management. 3. Patients involve their social network in the treatment.	**Social network is a bit important** Most patients (*n* = 5) shared that they had the main responsibility in their lifestyle or medication, although others (*n* = 2) said their social network was mainly responsible. Patients (*n* = 3) did not always see the need to involve their social network in the treatment.	**Social network is really important** Half of the patients (*n*=6) indicated that a significant other was mainly in the lead in lifestyle or medication, although others (*n* = 2) said they had no support in their self-management. Some said that HCPs do not involve social networks enough (*n* = 4).

CKD = chronic kidney disease, LHL = limited health literacy, HCP = health care professional, GP = general practitioner, *n* = number of interviewed patients talking about this experience. Experiences that indicate an important difference between ambulatory and dialysis setting are in bold.

**Table 3:** Evaluation of the intervention with patients and healthcare professionals.

Patients (*n* = 4)		Healthcare Professionals (*n* = 17)	
**Grade for intervention ^#^** mean ± stdev	7.75 ± 0.957	**Grade for intervention ^#^** mean ± stdev	7.97 ± 0.910
**Usability of the intervention (*n*)** ⋅ Complicated ⋅ Neutral ⋅ Easy	0 1 3	**Fit to daily practice (*n*)** Yes Partly No	17 0 0
**Complexity of the content (*n*)** ⋅ Complicated ⋅ Just right ⋅ Easy	0 3 1	**Complexity of workshop (*n*)** Complicated Just right Easy	0 12 5
**Length (*n*)** ⋅ Too long ⋅ Good ⋅ Too short	2 2 0	**Length (*n*)** Too long Good Too short	0 10 7
**Self-reported effect (*n*) on:** CKD understanding Understanding of consequences Understanding of lifestyle Lifestyle confidence Knowledge on consultation topics Consultation self-efficacy	4 3 1 1 3 2	Improved knowledge * mean ± stdev	5.94 ± 0.854
**Usefulness other patients ˆ** mean ± stdev	4.00 ± 0.000	Improved self-efficacy * mean ± stdev	5.75 ± 1.000
**Usefullness significant others ˆ** mean ± stdev	3.75 ± 0.500	Expected strategy use * mean ± stdev	6.13 ± 0.619

*n* = number of participants, stdev = standard deviation. ^#^ Rated between 1–10, as in the Dutch grading system; * Measured with 7-point Likert scales with statements. Answer options ranged from strongly disagree to strongly agree. Maximum possible score = 7. ˆ Measured with 5-point Likert scales with statements. Answer options ranged from strongly disagree to strongly agree. Maximum possible score = 5.

**Table 4:** Final logic model of change with the four components of our intervention, and final objectives, determinants and outcome expectations.

Objective	Determinants	Outcome Expectations	SocM ^#^
Improve awareness and knowledge on CKD self-management	HCPs know strategies to create CKD awareness in patients with LHL. HCPs inform CKD patients in simple language and with visual strategies. HCPs check the CKD patient’s understanding. Patients are aware of having CKD and what this diagnosis means (*). Patients understand (symptoms of) CKD and the long-term risks of CKD (*). Patients know important risk factors for developing more severe CKD (+). Patients know how self-management can stabilize kidney function (+). Patients ask for clarification and questions during consultations if needed.	Patients are more aware of CKD. Patients understand CKD better. Patients understand self-management of CKD better. Patients understand the long-term risks of CKD better. Patients feel more urgency to prevent further kidney deterioration. Patients discuss CKD better during consultations with HCPs.	Precontem-plation and contem-plation
Improve motivation and preparation of self-management	HCPs use health or life aims in goalsetting to motivate themselves to self-manage(+). HCPs apply shared decision making to decide on aims and self-management. Patients see the rewards of self-management for CKD and quality of life (*). Patients share their personal needs regarding self-management with HCPs. Patients prepare consultations to better discuss self-management (+). Patients feel confident to follow up self-management advice at home (+). Patients involve their social network in their self-management (*).	Patients know the exact goals of self-management of CKD. Patients contribute to decisions on self-management of CKD. Self-management goals are tailored to the patients’ needs. Patients are more confident to improve self-management. Patients are better able to adopt self-management in daily life. The social network helps to adopt self-management changes.	Preparation
Improve practical competences for self-management and to maintain behaviors on the long-term	HCPs translate general self-management advice into action points. HCPs ask about and respond to self-management barriers of the patient (+). HCPs seek solutions to barriers using shared decision making. Patients have the practical competences to improve lifestyle and medication adherence. Patients share their doubts regarding advice given by HCPs (+). Patients share their barriers and relapses with HCPs (+). Patients know strategies to prevent relapse of self-management changes. Patients recognize and solve barriers that negatively influence self-management (such as negative emotions, feasibility problems, relapse) (*). Patients seek additional help if they experience self-management barriers (+).	Patients gain practical skills for self-management of CKD. Patients are better at discussing barriers for self-management. Patients overcome barriers for maintenance of self-management. Patients maitain self-management changes in the long term. Patients deal better with emotions, infeasibility of advice and relapse. The social network supports patients in maintaining changes.	Action and maintenance
Improve the competences of HCPs	HCPs have awareness and knowledge of HL and its consequences. HCPs apply strategies to identify patients with LHL. HCPs involve the social network in consultation and treatment. HCPs empower the social network to contribute to self-management. HCPs know and apply tailored strategies to support patients with LHL during different stages of behavior change. These strategies are indicated behind the objectives above (informing patients in simple language, check understanding, using health or life aims, applying shared decision making, translating advice into action points, responding to barriers etc.) (*)(^~^).	HCPs have awareness and knowledge regarding health literacy. HCPs recognize patients with LHL. HCPs know effective strategies to support patients with LHL better and to involve the social network. HCPs apply the mentioned strategies effectively to support the patient during different stages of behavior change.	HCP support

^#^ SoCM = Stages of Change Model. * = adapted determinant, based on step 2 of the IM protocol. + = new determinant, based on step 2 of the IM protocol. ^~^ = to effectuate the patient-targeted objectives, the HCP plays an important role, as is visible in the determinants. The fourth objective targeting the HCPs aims to help them to acquire the needed strategies to support patients with LHL better. HCPs = health care professionals. LHL = limited health literacy.

## Data Availability

The data presented in this study are available on request from the corresponding author. The data are not publicly available as we only asked informed consent from the participants to share data with other researchers on request.

## Supplementary Materials

The following are available online at https://www.mdpi.com/article/10.3390/ijerph182413354/s1, Table S1: Identified barriers and proposed solutions to optimize self-management of CKD patients with LHL, Table S2. Quotations, derived from the determinants, which we discussed with patients, Table S3. Example of the form to collect experiences regarding the determinants from the interviews, Table S4. Objectives and determinants for the Health Liteacy Communication Training of Kaper et al, Table S5: Rating of the HL intervention for patients with the checklists of Rudd et al.
