# Supplementary material for: Co-Creation of a Multi-Component Health Literacy Intervention Targeting Both Patients with Mild to Severe Chronic Kidney Disease and Health Care Professionals
Source: Int J Environ Res Public Health. 2021 Dec 18;18(24):13354. doi: 10.3390/ijerph182413354 (PMC8704507; doi:10.3390/ijerph182413354)
Supplement: Supplementary file 1 [file ijerph-18-13354-s001.zip › ijerph-1411732-supplementary.pdf]

Supplementary Files:

Table S1. Identified barriers and proposed solutions to optimize self-management of CKD patients with LHL.

Identified Barriers for Self-Management	Proposed Solution
Patients with mild to moderate CKD[1] are often unaware of the existence and seriousness of their disease. HCPs[2] pay limited attention to CKD and mainly explain it as 'a value from the lab' without teaching self-management skills.	Improve CKD awareness in patients with mild to moderate CKD.
Patients lack knowledge regarding CKD and self-management and competences to retrieve information from documents or consultations. HCPs mainly provide information orally or written, which is not always comprehensible, and do not consistently check the patient's understanding of the disease and treatment.	Provide simple and visually attractive information and check patient understanding
Patients struggle to translate HCP's advices into daily life self-management. HCPs overestimate patients, focus too much on medical theory and the aims of treatment, without providing actionable information.	Provide practical step-wise guidance to facilitate self-management.
Patients see few or only short-term health benefits of self-management. They have problems to see why self-management is important. HCPs often motivate patients by emphasizing the importance of 'being healthy', while for patients life aims seem more important as the kidneys will never get better.	Motivate patients by using life aims and explaining the benefits of self-management better.
Patients mention barriers to maintain self-management on the long-term. They often attempt to change, but fail to persist when advises are unfeasible, they feel depressed or stressed, notice the kidneys are still declining, or fall for unhealthy food or habits. HCPs pay limited attention to these long-term self-management barriers.	Support patients to overcome long-term self-management barriers.
Patients mention the social network is important to start and maintain self-management. HCPs state the social network can either support or counteract self-management. Involvement of the network in the treatment is often insufficient.	Involve the social network to support the patient.
Patients and HCPs mention how strategies to improve self-management do not always meet the patient's needs. HCPs mention that they have problems to recognize HL[3] problems and to truly understand barriers of patients with LHL[4].	Train the professional to recognize and support patients with LHL.

The stated barriers and proposed solutions above were synthesized from in-depth interviews with CKD patients with LHL (n = 24) and focus group discussions and interviews with HCPs (n = 37) and are in the result section of our previous longitudinal, qualitative study [16]. We included them here as well, as they are the starting point of our intervention development. 1 CKD = chronic kidney disease, 2 HCP = health care professional, 3 HL = health literacy, 4 LHL = limited health literacy.

Table S2. Quotations, derived from the determinants, which we discussed with patients.

Determinants	Quotations for Patient Interviews[#]
Patients are aware of having kidney problems<br>HCPs know strategies to create CKD awareness in patients with LHL	'I am or was not aware of having kidney problems'
Patients understand (symptoms of) CKD<br>Patients know what the risks are, when they develop more severe CKD<br>Patients ask clarification from the HCP if needed<br>HCPs inform CKD patients in simple language and with visual strategies<br>HCPs check the CKD patients' understanding	'I understand my CKD'<br>'I doubt if it is dangerous when my kidney problems worsen'<br>'I find it difficult to ask for clarification'<br>'The explanation of the HCP can be too difficult'
Patients are intrinsically motivated to self-manage their disease and treatment<br>Patients share their personal needs regarding self-management with HCPs<br>HCPs apply shared-decision making to decide on aims and self-management	'I don't know what I will gain with lifestyle or medication'<br>'I don't know exactly what is important to tell the HCP'<br>'It is mainly the HCP telling me what I need to do to protect my kidneys. I will not speak up if I have other thoughts'
Patients have the practical competences to improve lifestyle and medication<br>HCPs translate general self-management advices into action points<br>HCPs respond to problems of the patient with advices to improve them	'Advises on lifestyle and medication are too generic. I miss how to practice them at home'<br>'The HCP is helping me to solve my practical problems with lifestyle or medication intake'
Patients recognize and solve barriers that negatively influence their self-management (such as negative emotions, feasibility problems)<br>Patients know strategies to prevent relapse of self-management changes<br>Patients share their barriers, concerns and relapse with HCPs.<br>HCPs seek for solutions for barriers, by applying shared decision making	'I often start to improve my lifestyle, but it is difficult to maintain'<br>'If needed, I will find solutions if things are going wrong'<br>'I do not always share my problems regarding lifestyle and medication with the HCP'
Patients involve the social network in the consultation and treatment<br>Patients seek additional help in the social network if they experience barriers<br>HCPs involve the social network in the consultation and treatment<br>HCPs empower the social network to contribute to self-management	'A significant other is responsible for my diet or medication'<br>'If I experience mental or physical problems or feel uncertain, I seek additional help'<br>'My HCP involves my significant others, for example in consultations'

[#] We selected a feasible amount of quotations to prevent that we asked too much of the patient. Therefore the number of quotations differs from the objectives. CKD = chronic kidney disease, HCP = health care professional.

Table S3. Example of the form to collect experiences regarding the determinants from the interviews.

Participant ID	CPD1
**Experiences related to determinants under awareness and knowledge**	Has been a kidney patient for years and has gradually learned quite a bit. Knows how to explain what the kidneys are and because of multiple transplants and years of dialysis, he is well aware of the risks. When he reads from the screen, I notice a lot of difficulty with written language. Something that is also confirmed by the questionnaire. Knows what is expected of him in lifestyle and can also name products that contain bad nutrients. Also good in understanding lab results.
**Preference for intervention strategies related to improving awareness and knowledge**	Finds it difficult to determine the type of intervention. On the one hand, his sister helps him with many things and it would be good for her to get information about illness and treatment together. On the other hand, he also finds autonomy important and sometimes wants to go to the cafe after a conversation. Alone or in pairs. Feels less for a group. He only shares problems with friends or family. Is enthusiastic abou the video and listens attentively. After watching, he indicates that a lot is correct in the video and it is a good way of informing in his eyes.
**Recognition of determinants related to communication with HCPs about the kidneys, risks and self-management preparation**	Does not recognize the communication problems very strongly. Thinks that he discusses many of his complaints with the doctor and also knows what is important. His example, neck pain, which he just talked about extensively with the nurse practioner, is not really a symptom of kidney problems, and brings up the question if he really knows important symptoms. Indicates that the doctor determines what is important to do in self-management and that you should not contradict it, because the doctor knows best. Doesn't always benefit from the advice about lifestyle, because he can't manage it that way. Would also like his network to be included in what needs to be done, because things often go differently or wrong when he tries.
**Preference for intervention strategies related to communication about the kidneys, risks and self-management preparation**	Reads the cartoon about asking clarification to the doctor, but finds it too complicated. Could also have been explained in one picture. Doctor saying a difficult word. Patient responding with I don't get it. Is very interested in the A4 sheets with icons with symptoms. Also handy to have this at home so he can show to others what he has. A lot of misunderstanding in the environment. Card to fill in, he immediately puts it into practice. Starts writing and ticking. The advice side seems useful to him to show to his environment and not to forget what was said during a consultation.
**Recognition of determinants about preparing and maintaining self-management at home and the approach of HCPs and network to support that**	From his story come many examples of the difficulty of self-management. Eating Chinese food, drinking beers, smoking, little exercise. Has difficulty with balancing having a nice life with lifestyle adherence, especially for diet. When a docor says he sometimes can live unhealthy, he does it probably too often. Also does not get the needed help from this network. Wants to walk with others, but, living in a sheltered home, he finds his supervisor stubborn and unhelpful. That makes him angry: they are there to help him with the things he finds difficult. He shares that he cannot come up with the solutions himself to problems he encounters.
**Preference for intervention strategies related to preparing and maintaining self-management and the role of HCPs and the social network**	Is quite outspoken in what he needs. A walking buddy. Also thinks it can benefit from reminders, for example to be reminded about the advices or to help to find solutions. Via WhatsApp for example. Does use the Internet to search or do things, so would be comfortable with digital solutions as well.
**Additions**	No additional comments.

Table S4. Objectives and determinants for the Health Liteacy Communication Training of Kaper et al.

**Objective A. To inform and educate: Professionals know about health literacy problems, their impact, and interventions to tackle health literacy problems**
1. Knowledge and awareness of health literacy<br>- Definition and overview of health literacy [29–48]<br>- Prevalence and risk factors of limited health literacy [29–32,34–36,40,48,49]<br>- Relation of health literacy to health outcomes [29–35,46,49,50]<br>- Cues to identify low health literacy [29–37,40,42,51]<br>- Formal identifiers of health literacy [34,35,38,39,41,42,45,48,49]<br>- Impact of limited health literacy on patients [29–32,34,36,39,41–45,49,50,52]
**Objective B. To teach skills: Professionals develop patient-centred communication skills to address problems with health literacy.**
2. Gathering and providing information to address functional health literacy.<br><br>Gathering information<br>- Active listening [32,42,53,54]<br>- Observing non-verbal communication [32,35,53,54]<br>- Asking open-ended questions [32,37,47,53–55]<br>- Encouraging patients to ask questions [32,39,42,47,53–55]<br>- Create a shame-free environment and responding to emotions [29,39,40,42,43,47,53,55]<br><br>Providing information<br>- Communicate clearly through plain language, avoidance of jargon, prioritization of information [29–35,37–40,42,44,47–49,51,55–57] - Using teach-back to check understanding [34,37–39,42,47,49,50,52,56,57]<br>- Assess and write comprehensible patient information [29–31,34,35,39–41,47–49,51] - Show or draw simple pictures [34,35,37,57]<br><br>3. Shared decision-making to address interactive health literacy.<br>- Involve patients in shared decision making [37,41,43,47,49,55,58–60]<br>- Educate patients to participate in shared decision-making [46,53,57,61].<br><br>4. Enabling self-management to address critical health literacy<br>- Discuss and facilitate patients' preparation for a consultation [53,59,62–65]<br>- Educate patients on self-management skills by repeating information and tailored education leaflets [33,39–41,45–47,53,56,57,59,62–68]<br>- Personal approach with exploring barriers to adherence, formulating treatment goals, co-design an action plan, monitor self-care [37,41,43,46,52,53,55,59,62–68] - Use (telephone) follow-up consultations to monitor understanding and self-care [33,47,53,55,56,59,62,64–67]
**Objective C. To support behaviour change: Professionals adopt, change and maintain behaviour to address health literacy problems**
5. Changing behaviour to apply health literacy communication<br>- Supporting behaviour change of professionals by influencing: Attitudes [69], Subjective norms [69] and self-efficacy [70]:<br>- Counselling low health literate patients [33,47,51]<br>- Practice based assignment [40,41,43–45]<br>- Feedback on clinical encounters with (standardised) patients [37,46,53,57,60].

Table S5. Rating of the HL intervention for patients with the checklists of Rudd et al.

Intervention Strategy	Mean Total Score	% (Range)
**Brochure (printed material)**		
Organization of information	1900	7917
Writing style and vocabulary	2533	9046
Design	2267	9446
Combined rating	6700	8816
**E-learning (website)**		
General features	1667	6944
Organization of information	2033	7262
Combined rating	3700	7115
**Consultation preparation card (form)**		
Combined rating	3000	7500
**Self-help booklet (printed material)**		
Organization of information	1567	6528
Writing style and vocabulary	1667	6944
Design	2233	9305
Combined rating	5467	7593

Irrelevant items, for example on math terms or abbreviations, which we did not use in our intervention, were excluded from the calculation.
